# Prognostic value of pulmonary congestion assessed by lung ultrasound imaging during heart failure hospitalisation: A two-centre cohort study

**DOI:** 10.1038/srep39426

**Published:** 2016-12-20

**Authors:** Stefano Coiro, Guillaume Porot, Patrick Rossignol, Giuseppe Ambrosio, Erberto Carluccio, Isabella Tritto, Olivier Huttin, Simon Lemoine, Nicolas Sadoul, Erwan Donal, Faiez Zannad, Nicolas Girerd

**Affiliations:** 1 Division of Cardiology, University of Perugia, School of Medicine, Via S. Andrea delle fratte, Perugia, Italy; 2 INSERM, Centre d’Investigations Cliniques 9501, Université de Lorraine, CHU de Nancy, Institut Lorrain du cœur et des vaisseaux, Nancy, France; 3 INI-CRCT (Cardiovascular and Renal Clinical Trialists) F-CRIN network, Nancy, France; 4 Departement de Cardiologie, CHU de Nancy, Institut Lorrain du cœur et des vaisseaux, Nancy, France; 5 Département de Cardiologie & CIC-IT U 804, Centre Hospitalier Universitaire de Rennes, France

## Abstract

Pulmonary congestion assessed at discharge by lung ultrasonography predicts poor prognosis in heart failure (HF) patients. We investigated the association of B-lines with indices of hemodynamic congestion [BNP, E/e’, pulmonary systolic arterial pressure (PAPs)] in HF patients, and their prognostic value overall and according to concomitant atrial fibrillation (AF), reduced (≤40%) ejection fraction (EF), and timing of quantification during hospitalisation for heart failure (HHF). In 110 HHF patients, B-lines were highly discriminative of BNP >400 pg/ml (AUC ≥ 0.80 for all), and moderately discriminative of PAPs >50 mmHg (AUC = 0.68, 0.56 to 0.80); conversely, B-lines poorly discriminated average E/e’ ≥ 15, except at discharge. B-line count significantly predicted mid-term recurrent HHF or death (overall and in subgroups), regardless of AF status, EF, and timing of quantification during HHF (all p for interaction >0.10). regardless, B-lines ≥30 at discharge were most predictive of outcome (HR = 7.11, 2.06–24.48; p = 0.002) while B-lines ≥45 early during HHF were most predictive of outcome (HR = 9.20, 1.82–46.61; p = 0.007). Lung ultrasound was able to identify patients with high BNP levels, but not with increased E/e’, also showing a prognostic role regardless of AF status, EF or timing of quantification; best B-line cut-off appears to vary according to the timing of quantification during hospitalization.

Clinical congestion is the most frequent cause in patients hospitalized for heart failure (HHF)^1^,^2^, and relieving congestion is a major goal of patients’ management during hospitalisation^3^. However, up to 25% of patients are discharged with residual congestion^2^. Clinical score on admission^4^ and at discharge^5^ have recently been shown to be of value for risk stratification. However, given the limited accuracy of physical examination in heart failure (HF) patients^6^, adding biological or imaging data may be helpful in improving risk stratification.

Lung ultrasound is a semi-quantitative, simple and reliable method to assess pulmonary congestion^7^, initially developed to diagnose alveolar-interstitial syndrome^8^. This technique produces images defined as “B-lines” (also known as “lung comets”) by scanning with probes along the intercostal spaces^9^. These artifacts are well associated with natriuretic peptides and echocardiographic indices of left ventricular (LV) filling pressures^10^, and seem to be determined by the degree of systolic and diastolic dysfunction and functional status assessed by New York Heart Association (NYHA) class^11^. Furthermore, lung ultrasound was shown to have a prognostic value on admission in an acute setting^12^,^13^; however, those studies were not specifically focused on HF patients. Conversely, as recently demonstrated by our group^14^ and others^15^, residual congestion quantified with B-lines is an independent predictor of short-term mortality^14^ and HF re-admission^14^,^15^ after hospitalisation for acute HF.

Brain natriuretic peptide (BNP), specifically secreted from cardiac chambers during acute decompensation^16^, have shown to be strong predictors of mortality on admission^17^ and at discharge^18^.

Echocardiography is the non-invasive gold standard currently used to evaluate LV filling pressures^19^, primarily using E/e’ ratio or other parameters following different algorithms according to ejection fraction (EF)^19^. However, in atrial fibrillation (AF) patients, this methodology has limitations due to lack of atrial contraction and presence of beat-by-beat alterations in preload^20^. Lung ultrasonography enables a straightforward congestion evaluation, less dependent on associated conditions (namely EF and AF) than echocardiographic assessment.

The aim of this study was to assess the discriminative value of B-lines in predicting high levels of biochemical and echocardiographic indices of hemodynamic congestion (i.e. BNP >400 pg/ml and E/e’ ≥ 15), and to determine its prognostic value in patients admitted for acute HF. These analyses were performed in the entire cohort, and according to the presence of (1) AF; (2) reduced ejection fraction (in absence of widely accepted criterion, we arbitrarily chose EF ≤ 40% as cut-off); (3) timing of quantification during hospital stay.

## Methods

### Participants

A total of 110 patients hospitalised for acute HF in two Cardiology departments (Division of “Cardiologia e Fisiopatologia Cardiovascolare”, Perugia, Italy and “Département de Cardiologie Medicale”, Nancy, France) were enrolled from April 3, 2014 to August 4, 2015. Inclusion criteria were: (1) age >18 years; (2) acute HF diagnosis, fulfilling the European Society of Cardiology criteria^3^, regardless of aetiology and systolic function. Exclusion criteria were: (1) pulmonary fibrosis, significant pleural effusion, severe emphysema, pleurisy, previous pneumectomy or lobectomy, pulmonary cancer or metastases, breast prosthesis; (2) pregnancy; (3) features potentially impacting follow-up (planned revascularisation, long distance between home and hospital).

In Perugia, the study was approved by the local ethics committee, complying with the Declaration of Helsinki^21^ and all patients provided written informed consent. In Nancy, the study was retrospective and lung echo data were acquired as part of usual clinical management; collection of patient files was approved by the Commission Nationale Informatique et Libertés (CNIL), in keeping with French law for single-centre usual care observational studies.

#### Echocardiographic-lung ultrasound study and natriuretic peptides assessment

All patients underwent echocardiography and lung ultrasound examination at rest using a Vivid 7 or a Vivid I (transducer 3S – RS) ultrasound system (GE HealthCare). LV volumes and EF (obtained using the biplane method of disks summation, the modified Simpson’s rule), inferior cava (IVC) diameter, and systolic pulmonary artery pressure (PAPs), were assessed according to current recommendations^22^. Diastolic function was assessed from the pattern of mitral inflow by pulsed Doppler echocardiography (E/A ratio); the examination was complemented by septal, lateral, and average mitral annular velocity measured by tissue Doppler imaging (E/e’ ratio)^19^. Lung ultrasound examinations were performed with patients in the supine or near-to-supine position as previously described^12^. Briefly, we scanned right and left haemithorax from the second to fourth (fifth on the right side) intercostal spaces, along four anatomical lines (parasternal, midclavicular, anterior axillary, midaxillary). The number of B-lines recorded in each of the 28 scanning site was summed, yielding a total score denoting the extent of pulmonary congestion, ranging from 0 to 280. (see Supplementary Materials, Figures S1–S3) B-lines were defined as reverberation artifacts starting from the pleural line and extending to the bottom of the screen without disappearing, and moving synchronously with pleural sliding^7^. All exams were performed by two operators (SC and GP) who did not take part in the clinical management at discharge of patients. Typically, all LUS were performed in 5 minutes, or less. Intra- and inter-observer reliability of lung ultrasound examinations were assessed by the two operators on the recordings of 30 loops, showing intra- and inter-observer correlation coefficients of 0.95 and 0.85, respectively.

Lung ultrasonographies were performed on the day of discharge in Perugia and during the first 3 days following admission in Nancy, at the end of the standard two-dimensional echocardiogram. Peripheral venous blood samples for BNP analysis were obtained on the day of the echocardiographic assessment except in 9/60 patients evaluated at discharge; in these small subset of patients, data obtained within the 2 days prior to discharge were used.

#### Decision of the timing of discharge and discharge drug therapy

In Perugia, all study-specific activities were performed independently of usual patient work-up; conversely, in Nancy, ultrasonographies were performed during the early management of patients. In both centres, treating physicians who were in charge of the patient at discharge had no knowledge of B-line results; discharge timing was based on a comprehensive clinical, instrumental and biochemical evaluation. All patients were on stable oral diuretic regimens for at least 24 h before discharge^3^.

#### Statistical methods

Continuous variables are expressed as mean (±standard error) or as median (inter-quartile range); categorical variables are presented as counts and percentages. Univariable comparisons in the three pairs of groups (according to AF, EF ≤40% and timing of quantification) were performed using chi-square or 2-sample Student t-test, as appropriate.

Receiver-operating characteristic (ROC) curves with BNP >400 pg/ml^16^ and average E/e’ ≥ 15^19^ (reference values for elevated left ventricular filling pressure) as well as with PAPs >50 mmHg (reference for reliably identifying pulmonary hypertension)^23^ were plotted. Outcome assessed during follow-up was a composite of HF hospitalisation or death at three months, established according to prior definitions^24^. Univariable analyses by Cox proportional hazard models were performed to assess the associations between B-lines and the considered outcome. B-lines were tested both as continuous and as categorical covariates, evaluating different cut-off levels previously found as having a prognostic value in this setting (i.e. B-lines ≥30^14^,^15^ and B-lines ≥15^14^) as well as values not yet tested (B-line s ≥ 45). Multivariable analyses were performed adjusting each B-line variable for a model including other indices of haemodynamic congestion (BNP, IVC diameter and E/e’ ≥ 15) and other variables considered a priori as relevant in this setting (NYHA class ≥III)^14^. Univariable and multivariable Cox proportional hazard models were performed both in the whole cohort and in subgroups, in addition to testing for interaction between B-lines and the three stratifying variables. The Kaplan-Meier method was used to estimate survival probabilities for the outcome in the entire cohort and in subgroups according to the different aforementioned cut-offs using the log-rank test to analyse differences.

A probability value < 0.05 was considered significant. Statistical analyses were performed using SPSS package version 23.0 (Chicago, Illinois).

## Results

### Patients’ characteristics

One hundred and ten patients were included in the present cohort (Table 1), 55% of whom were male, with an overall mean age of 73 ± 1 years and a mean EF of 39 ± 1%.

Patients with EF ≤40% had higher median BNP values than patients with a higher EF (1132 vs. 415 pg/ml, p < 0.0001). Patients with AF had longer hospital stay (8 vs 6 days, p = 0.004) than patients without AF, as well as lower estimated glomerular filtration rate (59 ± 4 vs. 68 ± 5 ml/min/1.73 m2, p = 0.013) and larger IVC diameters (22 ± 1 vs. 19 ± 1 mm, p = 0.002).

Our cohort included 60 discharge patients and 50 patients enrolled during hospitalisation (among the latter subgroup, 17 were patients enrolled the day of admission, with the remaining evaluated on average at 1.9 days). As expected, discharge patients had lower median BNP levels (574 vs. 765 pg/ml, p = 0.012), smaller IVC diameters (20 ± 1 vs. 22 ± 1 mm, p = 0.002), lower B-line counts (23 ± 3 vs. 47 ± 4, p < 0.0001), and less likely to be in NYHA class ≥III (15% vs. 84%, p < 0.0001) (Table 1).

### Discriminative value of B-lines for average E/e’, BNP and pulmonary artery systolic pressure

**Figure 1:**
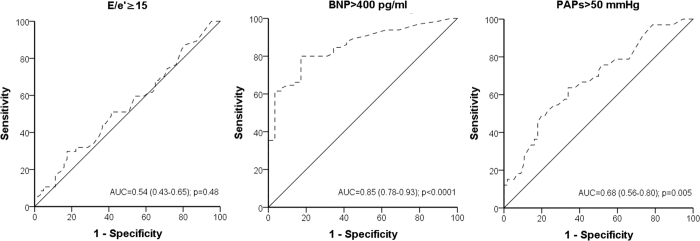
ROC curves for (B-lines, lung comets) and average E/e’ ≥ 15 (panel A), BNP >400 pg/ml (panel B) and PAPs >50 mmHg (panel C) in the whole cohort.

B-lines were poorly discriminative in predicting E/e’ ≥ 15, with an area under the curve (AUC) of 0.54 (95% CI: 0.43 to 0.65, p = 0.48) for the entire cohort (Fig. 1A) as well as for EF and AF subgroups (Table 2). Conversely, there was a good discriminative value between B-lines at discharge and E/e’ ≥ 15, with an AUC of 0.76 (95% CI 0.63–0.87, p = 0.001) as opposed to patients with B-lines during hospitalisation (AUC = 0.61, 95% CI 0.43–0.78, p = 0.292) (Table 2).

B-lines showed a good discriminative value in predicting BNP >400 pg/ml in the entire cohort (AUC = 0.85, 95% CI: 0.78 to 0.93, p < 0.0001) (Fig. 1B), with optimal cut-off at 21 B-lines (both sensitivity and specificity equal to 80%). Subgroup analysis confirmed a good discriminative value in all subgroups (Table 2). With specific regard to subgroups stratified according to discharge assessment, the optimal cut-off in predicting discharge BNP >400 pg/ml was at 9 B-lines (with both sensitivity and specificity of 78%); for B-lines assessed during hospitalisation, the optimal cut-off in predicting this reference parameter was at 29, value associated with a 78% sensitivity and a 76% specificity.

B-lines showed a fair discriminative value in differentiating patients with pulmonary hypertension (AUC = 0.68, 0.56 to 0.80, p = 0.005) (Fig. 1C); presence of 27 B-lines was found to maximise the overall diagnostic accuracy with a sensitivity of 64% and a specificity of 63%. B-lines similarly discriminated pulmonary hypertension in the other subgroups (Table 2).

### Follow-up data: mortality and HF hospitalisation

At 3 month follow-up, a total of 33 combined events occurred, with 26 HF hospitalisations and 16 deaths.

Univariable analysis showed that B-lines as continuous variable (tested for each 10 incremental units) was a significant predictor for the outcome in the whole cohort as well as in all subgroups (Table 3). By multivariable analysis, B-lines remained a significant predictor in the whole cohort (HR = 1.36, 1.14 to 1.61, p = 0.001) as well as in all the subgroups, without evidence of interaction across subgroups (Table 3).

Among the three tested cut-off counts, the most relevant results were found for B-lines ≥45 and B-lines ≥30 (Table 3). In multivariable analysis, both B-lines ≥45 (HR = 4.60, 1.73 to 12.25, p = 0.002) and B-lines ≥30 (HR = 6.08, 1.97 to 18.75, p = 0.002) were significant predictors in the whole cohort as well as in patients with AF or in patients with EF >40% (Table 3). Of note, with regard to the timing of lung ultrasound assessment, B-lines ≥45 was a significant predictor only for patients assessed during hospitalisation (HR = 9.20, 1.82 to 46.61, p = 0.007) but not for those assessed at discharge. In contrast, B-lines ≥30 was significantly associated with the combined outcome only for discharge patients (HR = 7.11, 2.06 to 24.48, p = 0.002) (Table 3). B-lines ≥15 was, in general, a significant predictor only by univariable analysis (Table 2).

Interaction between all B-line variables and each of three aforementioned stratifying variables (AF status, EF, and timing of B-line quantification during hospitalisation for HF) were tested, none of which were significant (Table 3).

**Figure 2:**
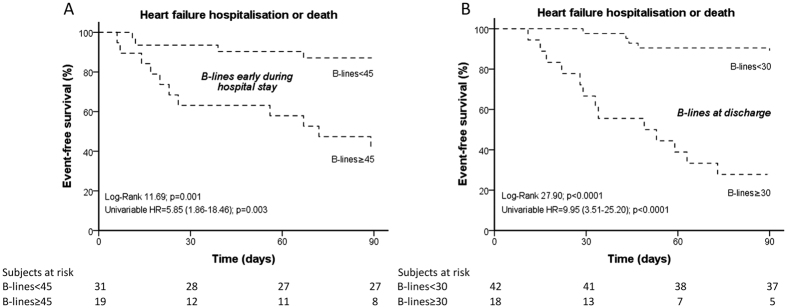
Kaplan Meier survival curves for HF hospitalisation or death in patients with either B-lines ≥45 or B-lines <45 assessed during HF hospitalisation (panel A) and with either B-lines ≥30 or B-lines <30 assessed at discharge (panel B).

For patients assessed at discharge, the 3-month risk for HF hospitalisation or death was 73% ± 10 in patients with B-lines ≥30 and 12% ± 5 in those with B-lines <30 (p < 0.0001); for patients assessed during hospitalisation, risk for the combined endpoint conferred by B-lines ≥45 was 58% ± 11, while B-lines <45 conferred a 13% ± 6 risk (p = 0.001) (Fig. 2A and B).

## Discussion

Pulmonary congestion assessed by lung ultrasound is an independent predictor of short-term mortality and HF hospitalisation regardless of AF status, EF or timing of quantification during hospitalization. Importantly, the most appropriate B-line count cut-off appeared to vary according to the timing of assessment (B-lines ≥45 for the early phase of HF hospitalisation and B-lines ≥30 at discharge). Furthermore, B-lines were found to have a good discriminative value in identifying patients with high BNP levels, but not with increased E/e’.

### Association between lung ultrasound data, BNP and filling pressures

Our study confirms the good association between B-lines and BNP, consistent with reports in HF outpatients^10^, corroborating these findings in the entire cohort and in subgroups.

Nevertheless, at variance with what observed in a chronic setting^10^, when using as reference E/e’ ≥ 15, B-lines had a fair discriminative value only at discharge. This can be partly explained by technical difficulties that may arise in an acute setting; indeed, E/e’ assessment can be very challenging and mitral inflow Doppler velocity measurements are more likely to be less accurate (i.e. for an inappropriate placement of the sample volume due to respiratory state or tachycardia). In addition, patients assessed early during hospitalisation were more likely to have severe valvular disease. These patients may have a higher degree of mitral annular calcification, which may increase E velocity^25^ or decrease e’ velocity^19^,^25^. Furthermore, during the first days of recompensation, clearing of B-lines may not promptly follow E/e’ reduction, as opposed to discharge, when haemodynamic congestion estimated by tissue Doppler imaging reaches a steady-state. In patients developing acute HF, ‘pulmonary congestion’ follows increased pulmonary capillary wedge pressure (‘haemodynamic congestion’)^26^. While pulmonary congestion can resolve rapidly with treatment, haemodynamic congestion relieving may be delayed or incomplete despite aggressive diuretic therapy. In acute HF patients followed for up to 24 h, (before and after intravenous diuretic treatment), improvement in clinical status is simultaneous and consistent with reduction in BNP levels, PAPs and number of B-lines; conversely, changes in E/e’ appear to be negligible^27^. Further studies assessing kinetics of B-lines and other congestion indices at regular intervals during a HHF are warranted. Lastly, the on-going EURO-FILLING study should provide greater insight on the effective accuracy of E/e’ ratio in estimating LV filling pressure^28^, validating this non-invasive parameter against left heart cardiac catheterisation by studying a large cardiac population with and without HF^28^.

### Ultrasonographic assessment of congestion and its prognostic value

In the setting of chronic HF, there is robust evidence demonstrating that an echocardiographic score based on indirect indicators of left ventricular intra-cavity filling pressure could be of value in predicting outcome^29^. Assessment of IVC and E/e’ have also been reported as independent predictors of poor outcome in HF outpatients^30^, and inpatients^31^, respectively.

We further confirm that lung ultrasound is a reliable prognostic tool, extending previous observations in an acute setting^12^,^13^,^14^,^15^. An increment of 10 B-lines conferred a considerable increased risk of adverse outcome (in entire cohort and subgroups). More importantly, B-lines ≥45 and B-lines ≥30 predicted the combined endpoint during the first days of HF hospitalisation and at discharge respectively, thus expanding our previous findings focused on discharge assessment^14^.

Gargani *et al*.^15^ recently evaluated B-lines prognostic value at short-term in acute HF patients. The average amount of B-lines on admission and at discharge was 48 and 20, which is perfectly in keeping with our findings (47 and 23 respectively); this confirms that lung ultrasound is a highly reproducible tool in this setting. Furthermore, among the tested cut-offs in that study, only B-lines ≥15 significantly predicted 6 months HF hospitalisation, while B-lines ≥50 at admission tended to be associated with outcome (p = 0.07)^15^; in our cohort, B-lines ≥15 at discharge tended to be associated with outcome (p = 0.055). Considering the different predetermined endpoints and length of follow-up, results from these two studies are very comparable.

### Clinical implications

Lung ultrasound can easily be performed throughout the course of in-hospital management of acute HF, thanks to portability of recently introduced hand-held devices; this technique allows for an extension of the physical examination by recognising, quantifying and monitoring pulmonary congestion at patient bedside in real-time, also improving risk-stratification over clinical and biological data. The 28-site method we used is a swift (performed in less than 5 minutes) tool to quantify the degree of congestion in HF patients, and is more accurate than the 8-zone method. We do believe that B-lines are, as reported by others^32^, ‘the kindergarten of echography’ and have tremendous potential to become the extension of the clinical examination in patients hospitalized for acute HF during routine bedside evaluation^33^, the ‘superstethoscope’ of the future^34^. More importantly, this new tool may also prove valuable in tailoring treatment, especially for in-hospital diuretics and possibly aquapheresis^35^. The practicability of lung ultrasound performed by hand-held devices and the high prognostic value of pulmonary congestion could render B-lines a highly useful therapeutic target in patients hospitalised for worsening HF. For example, a strategy based on B-line evaluation could help toward better decongestion in these patients, and thus translate in better mid-term outcomes. Such strategy would obviously have to be formally tested in future randomised clinical trials.

## Limitations

The follow up period was relatively short. A further larger independent multicentre study is warranted to confirm the strong association observed herein between B-line counts and outcome. Moreover, B-lines were detected at different stages in the two centres (in Nancy during early stage and in Perugia at discharge), representing an important limitation; however, B-line counts were perfectly in line with previously single-center studies^15^.

B-line detection does not necessarily imply their cardiogenic origin as they are a very sensitive but albeit nonspecific sign of pulmonary congestion. However, when assessing patients with an already established diagnosis of acute HF as the case in the present study, this issue is less relevant. The clinical context is the key to avoid gross misinterpretation of this sign; indeed, when a large number of B-lines are totally unrelated to the clinical picture, caution should be used and other causes of B-lines should be taken into account.

B-lines can be found in a number of other pulmonary conditions, such as pneumonia, atelectasis and acute lung injury/acute respiratory distress syndrome, hence implying a differential diagnosis that was not present in our study population.

In addition, although the investigators who performed B-lines assessment had knowledge about clinical status and echocardiographic data, all decisions regarding timing of discharge were taken by the cardiologist who was unaware of B-line data. Lastly, real-time analysis may represent a potential source of bias. However, real-time analysis could ultimately become highly prominent in the clinical setting if B-line recordings were to become routine care.

## Conclusion

Pulmonary congestion assessed by lung ultrasound throughout acute HF hospitalisation is a strong predictor of short-term HF re-admission or death. Additionally, lung ultrasound assessment during hospitalisation for HF represents a useful tool to identify and monitor congestion, given its ability to discriminate haemodynamic congestion status, especially when using natriuretic peptides and PAPs as reference landmarks. Lung ultrasound, given its practicability, simplicity and reproducibility even when used by beginners, could become the extension of clinical examination in patients with acute HF. Clinical trials assessing whether this tool could ultimately guide therapy optimisation are certainly worthwhile.

## Additional Information

**How to cite this article:** Coiro, S. *et al*. Prognostic value of pulmonary congestion assessed by lung ultrasound imaging during heart failure hospitalisation: A two-centre cohort study. *Sci. Rep.* **6**, 39426; doi: 10.1038/srep39426 (2016).

**Publisher's note:** Springer Nature remains neutral with regard to jurisdictional claims in published maps and institutional affiliations.

## Supplementary Material

Supplementary Information

## Figures and Tables

**Table 1:** Study population characteristics in the whole cohort and according to atrial fibrillation status, ejection fraction and timing of B-lines quantification.

Variables	All patients (N = 110)	EF ≤ 40% N = 59 (54%)	EF >40% N = 51 (46%)	P- value	AF yes N = 64 (58%)	AF no N = 46 (42%)	P- value	B-linequantification early during hospital stay N = 50 (45%)	B-line quantification at discharge N = 60 (55%)	P- value
Age (years)	73 ± 1	72 ± 2	73 ± 2	0.920	73 ± 2	71 ± 2	0.592	73 ± 2	72 ± 1	0.53
Male sex n (%)	60 (55%)	36 (61%)	24 (47%)	0.143	33 (52%)	27 (59%)	0.459	19 (38%)	41 (68%)	0.001
Length of stay (day)	7 (9)	7 (9)	7 (8)	0.302	8 (10)	6 (5)	0.004	10 (9)	5 (4)	<0.0001
Ischaemic aetiology n (%)	50 (46%)	26 (44%)	24 (47%)	0.753	29 (45%)	21 (46%)	0.972	31 (62%)	19 (32%)	0.001
Valvular heart disease n (%)	36 (33%)	17 (29%)	19 (38%)	0.339	20 (32%)	16 (35%)	0.783	23 (46%)	13 (21%)	0.004
Previous HF admission n (%)	79 (72%)	39 (67%)	40 (82%)	0.156	57 (92%)	22 (51%)	<0.0001	34 (76%)	45 (75%)	0.948
eGFR MDRD (ml/min)	62 ± 3	62 ± 4	63 ± 4	0.975	59 ± 4	68 ± 5	0.013	62 ± 6	63 ± 3	0.812
BNP (pg/ml)	709 (1089)	1132 (1211)	415 (514)	<0.0001	683 (1358)	749 (838)	0.559	765 (1270)	574 (918)	0.012
EF (%)	39 ± 1	27 ± 1	54 ± 1	<0.0001	40 ± 2	41 ± 3	0.607	37 ± 2	42 ± 2	0.122
IVC (mm)	21 ± 1	21 ± 1	20 ± 1	0.110	22 ± 1	19 ± 1	0.002	22 ± 1	20 ± 1	0.026
B-lines	32 ± 2	40 ± 4	24 ± 4	0.036	32 ± 4	32 ± 4	0.185	47 ± 4	23 ± 3	<0.0001
B-lines ≤15	39 (35%)	19 (32%)	20 (39%)	0.443	19 (30%)	20 (43%)	0.136	2 (4%)	37 (72%)	<0.0001
B-lines ≤30	57 (52%)	26 (44%)	31 (61%)	0.080	30 (47%)	27 (59%)	0.221	20 (30%)	42 (70%)	<0.0001
B-lines ≤45	82 (74%)	40 (68%)	42 (82%)	0.081	44 (69%)	38 (83%)	0.100	31 (62%)	51 (85%)	0.006
Average E/e’	16 ± 1	19 ± 1	15 ± 1	0.185	16 ± 1	18 ± 2	0.458	15 ± 1	19 ± 1	0.105
NYHA ≥III n (%)	51 (46%)	30 (51%)	21 (41%)	0.310	34 (53%)	17 (37%)	0.093	42 (84%)	9 (15%)	<0.0001

Values are expressed as mean ± standard error for all continuous variables except BNP and length of stay, expressed as median (interquartile range); values for categorical variables are expressed as number of cases (%). NYHA = New York Heart Association; eGFR = Estimated glomerular filtration rate; MDRD = modification of diet in renal disease; EF = ejection fraction; BNP = brain natriuretic peptide; IVC = inferior vena cava, AF = atrial fibrillation.

**Table 2:** Discrimination value of B-lines in predicting average E/e’ ≥ 15, BNP >400 pg/ml and PAPs >50 mmHg.

	AUC (95% C.I.)	p value		AUC (95% C.I)	p value		AUC (95% C.I)	p value
**B-lines/E/e’ average ≥15**			**B-lines/BNP >400**			**Comets-PAPs >50**		
**Overall (N = 110)**	0.54 (0.43–0.65)	0.48	**Overall (N = 94)**	0.85 (0.78–0.93)	<0.0001	**Overall (N = 89)**	0.68 (0.56–0.80)	0.005
**AF**			**AF**			**AF**		
**Yes (N = 64)**	0.48 (0.34–0.63)	0.87	**Yes (N = 54)**	0.84 (0.73–0.94)	<0.0001	**Yes (N = 51)**	0.68 (0.51–0.82)	0.048
**No (N = 46)**	0.62 (0.46–0.79)	0.14	**No (N = 40)**	0.91 (0.83–1)	<0.0001	**No (N = 38)**	0.68 (0.50–0.85)	0.063
**EF**			**EF**			**EF**		
**≤40% (N = 59)**	0.48 (0.33–0.63)	0.86	**≤40% (N = 48)**	0.90 (0.80–0.99)	0.001	**≤40% (N = 49)**	0.73 (0.58–0.88)	0.008
**>40% (N = 51)**	0.57 (0.39–0.74)	0.42	**>40% (N = 46)**	0.80 (0.65–0.92)	0.001	**>40% (N = 40)**	0.64 (0.46–0.81)	0.142
**Timing of B-lines quantification**			**Timing of B-lines quantification**			**Timing of B-lines quantification**		
**Early during hospital stay (N = 50)**	0.61 (0.43–0.78)	0.292	**Early during hospital stay (N = 43)**	0.86 (0.73–0.99)	0.001	**Early during hospital stay (N = 29)**	0.74 (0.56–0.93)	0.027
**At discharge (N = 60)**	0.76 (0.63–0.87)	0.001	**At discharge (N = 51)**	0.84 (0.73–0.95)	<0.0001	**At discharge (N = 60)**	0.60 (0.44–0.75)	0.25

Discrimination value of B-lines in predicting average E/e’ ≥ 15, BNP **>**400 pg/ml and PAPs **>**50 mmHg in the whole cohort and in subgroups stratified according to ejection fraction (EF ≤ 40%), atrial fibrillation and discharge B-line assessment.

AUC = area under the curve; BNP = brain natriuretic peptide; EF = ejection fraction; PAPs = pulmonary artery pressure, systolic.

**Table 3:** Univariable and multivariable analyses for the combined endpoint of heart failure hospitalisation and/or death.

	B-lines (continuous)			B-lines ≥45			B-lines ≥30			B-lines ≥15		
	HR	95% C.I.	p value	HR	95% C.I.	p value	HR	95% C.I.	p value	HR	95% C.I.	p value
**Univariable analysis**												
Overall	1.32	1.15–1.47	<0.0001	5.12	2.57–10.20	<0.0001	5.05	2.19–11.65	<0.0001	3.74	1.44–9.69	0.007
AF												
yes	1.21	1.05–1.41	0.011	4.17	1.82–9.53	0.001	3.11	1.23–7.90	0.017	1.77	0.65–4.75	0.26
no	1.46	1.78–1.82	0.01	6.79	1.96–22.53	0.003	16.30	2.06–28.21	0.009	/	/	/
Interaction	p = 0.165			p = 0.520			p = 0.152			p = 0.913		
EF												
≤40%	1.26	1.05–1.50	0.012	4.89	1.78–13.47	0.002	4.22	1.20–14.81	0.025	2.39	0.68–8.38	0.17
>40%	1.42	1.20–1.68	<0.0001	7.29	2.79–19.07	<0.0001	6.90	2.24–21.21	0.001	6.129	1.44–27.51	0.015
Interaction	p = 0.328			p = 0.573			p = 0.567			p = 0.328		
Timing of B-line quantification												
Early during hospital stay	1.37	1.18–1.60	<0.0001	5.85	1.86–18.46	0.003	3.08	0.69–13.63	0.139	5.63	2.01–15.84	0.001
At discharge	1.30	1.06–1.64	0.014	4.06	1.98–19.58	0.002	9.95	3.51–25.20	<0.0001	/	/	/
Interaction	p = 0.755			p = 0.972			p = 0.224			p = 0.929		
**Multivariable analysis**												
Overall	1.36	1.14–1.61	0.001	4.60	1.73–12.25	0.002	6.08	1.97–18.75	0.002	2.78	0.80–9.72	0.109
AF												
yes	1.35	1.09–1.71	0.008	5.54	1.75–17.57	0.004	3.82	1.11–13.12	0.033	2.54	0.59–10.99	0.214
no	1.41	1.08–1.84	0.012	3.99	0.86–18.61	0.290	/	/	/	/	/	/
EF												
≤40%	1.29	1.02–1.67	0.048	3.40	0.85–13.55	0.084	4.24	0.80–22.42	0.089	2.50	0.41–15.28	0.320
>40%	1.54	1.23–1.92	<0.0001	7.75	2.28–26.30	0.001	10.10	2.44–41.98	0.001	7.47	1.38–40.46	0.020
Timing of B-line quantification												
Early during hospital stay	1.48	1.06–2.06	0.021	9.20	1.82–46.61	0.007	3.12	0.37–26.19	0.295	/	/	/
At discharge	1.30	1.07–1.59	0.009	2.91	0.74–11.44	0.127	7.11	2.06–24.48	0.002	4.10	0.95–14.63	0.055

Univariable and multivariable analysis for B-lines, as continuous variable (per 10 B-lines of increment) and as categorical variable (3 cut-off values, B-lines ≥45, B-lines ≥30, B-lines ≥15) In multivariable analysis, B-lines were adjusted for other indices of haemodynamic congestion (BNP and average E/E’, both as continuous variables) and relevant *a priori* covariate (NYHA ≥3 and inferior vena cava diameter considered as a continuous variable). HR = hazard ratio; EF = ejection fraction; AF = atrial fibrillation; C.I. = confidence intervals./ = not applicable as the model did not converge.
