# Corrigendum: Prognostic value of pulmonary congestion assessed by lung ultrasound imaging during heart failure hospitalisation: A two-centre cohort study

**DOI:** 10.1038/srep43972

**Published:** 2017-03-16

**Authors:** Stefano Coiro, Guillaume Porot, Patrick Rossignol, Giuseppe Ambrosio, Erberto Carluccio, Isabella Tritto, Olivier Huttin, Simon Lemoine, Nicolas Sadoul, Erwan Donal, Faiez Zannad, Nicolas Girerd

Scientific Reports 6: Article number: 3942610.1038/srep39426; published online: 11 20 2016; updated: 03 16 2017

This Article contains errors in Table 3. In the “B-lines ≥15” column, the Timing of B-line quantification values using univariable analysis for “Early during hospital stay” and “At discharge” are inverted. The correct Table 3 appears below.

## Figures and Tables

**Table 3:** Univariable and multivariable analyses for the combined endpoint of heart failure hospitalisation and/or death.

	B-lines (continuous)			B-lines ≥45			B-lines ≥30			B-lines ≥15		
	HR	95% C.I.	p value	HR	95% C.I.	p value	HR	95% C.I.	p value	HR	95% C.I.	p value
**Univariable analysis**												
Overall	1.32	1.15–1.47	< 0.0001	5.12	2.57–10.20	< 0.0001	5.05	2.19–11.65	< 0.0001	3.74	1.44–9.69	0.007
AF												
yes	1.21	1.05–1.41	0.011	4.17	1.82–9.53	0.001	3.11	1.23–7.90	0.017	1.77	0.65–4.75	0.26
no	1.46	1.78–1.82	0.01	6.79	1.96–22.53	0.003	16.30	2.06–28.21	0.009	/	/	/
Interaction	p = 0.165			p = 0.520			p = 0.152			p = 0.913		
EF												
≤40%	1.26	1.05–1.50	0.012	4.89	1.78–13.47	0.002	4.22	1.20–14.81	0.025	2.39	0.68–8.38	0.17
>40%	1.42	1.20–1.68	<0.0001	7.29	2.79–19.07	<0.0001	6.90	2.24–21.21	0.001	6.129	1.44–27.51	0.015
Interaction	p = 0.328			p = 0.573			p = 0.567			p = 0.328		
Timing of B-line quantification												
Early during hospital stay	1.37	1.18–1.60	< 0.0001	5.85	1.86–18.46	0.003	3.08	0.69–13.63	0.139	/	/	/
At discharge	1.30	1.06–1.64	0.014	4.06	1.98–19.58	0.002	9.95	3.51–25.20	< 0.0001	5.63	2.01–15.84	0.001
Interaction	p = 0.755			p = 0.972			p = 0.224			p = 0.929		
**Multivariable analysis**												
Overall	1.36	1.14–1.61	0.001	4.60	1.73–12.25	0.002	6.08	1.97–18.75	0.002	2.78	0.80–9.72	0.109
AF												
yes	1.35	1.09–1.71	0.008	5.54	1.75–17.57	0.004	3.82	1.11–13.12	0.033	2.54	0.59–10.99	0.214
no	1.41	1.08–1.84	0.012	3.99	0.86–18.61	0.290	/	/	/	/	/	/
EF												
≤40%	1.29	1.02–1.67	0.048	3.40	0.85–13.55	0.084	4.24	0.80–22.42	0.089	2.50	0.41–15.28	0.320
>40%	1.54	1.23–1.92	<0.0001	7.75	2.28–26.30	0.001	10.10	2.44–41.98	0.001	7.47	1.38–40.46	0.020
Timing of B-line quantification												
Early during hospital stay	1.48	1.06–2.06	0.021	9.20	1.82–46.61	0.007	3.12	0.37–26.19	0.295	/	/	/
At discharge	1.30	1.07–1.59	0.009	2.91	0.74–11.44	0.127	7.11	2.06–24.48	0.002	4.10	0.95–14.63	0.055

Univariable and multivariable analysis for B-lines, as continuous variable (per 10 B-lines of increment) and as categorical variable (3 cut-off values, B-lines ≥45, B-lines ≥30, B-lines ≥15) In multivariable analysis, B-lines were adjusted for other indices of haemodynamic congestion (BNP and average E/E’, both as continuous variables) and relevant *a priori* covariate (NYHA ≥ 3 and inferior vena cava diameter considered as a continuous variable). HR = hazard ratio; EF = ejection fraction; AF = atrial fibrillation; C.I. = confidence intervals. / = not applicable as the model did not converge.

