# Supplementary material for: Prognostic value of pulmonary congestion assessed by lung ultrasound imaging during heart failure hospitalisation: A two-centre cohort study
Source: Sci Rep. 2016 Dec 20;6:39426. doi: 10.1038/srep39426 (PMC5171824; doi:10.1038/srep39426)
Supplement: Supplementary Information [file srep39426-s1.pdf]

Prognostic value of pulmonary congestion assessed by lung ultrasound imaging during heart failure hospitalisation: A two-centre cohort study.

Stefano Coiro, 1,2; Guillaume Porot, 3; Patrick Rossignol, 2; Giuseppe Ambrosio, 1; Erberto Carluccio, 1; Isabella Tritto, 1;Olivier Huttin, 3,Simon Lemoine, 3; Nicolas Sadoul, 3; Erwan Donal, 4; Faiez Zannad, 2, 3; Nicolas Girerd, 2, 3*

1 Division of Cardiology, University of Perugia, School of Medicine, Via S. Andrea delle fratte, Perugia, Italy

2 INSERM, Centre d'Investigations Cliniques 9501, Université de Lorraine, CHU de Nancy, Institut Lorrain du cœur et des vaisseaux, Nancy, France and INI-CRCT (Cardiovascular and Renal Clinical Trialists) F CRIN network, Nancy, France

3 Departement de Cardiologie, CHU de Nancy, Institut Lorrain du cœur et des vaisseaux, Nancy, France

4Département de Cardiologie & CIC-IT U 804, Centre Hospitalier Universitaire de Rennes, France

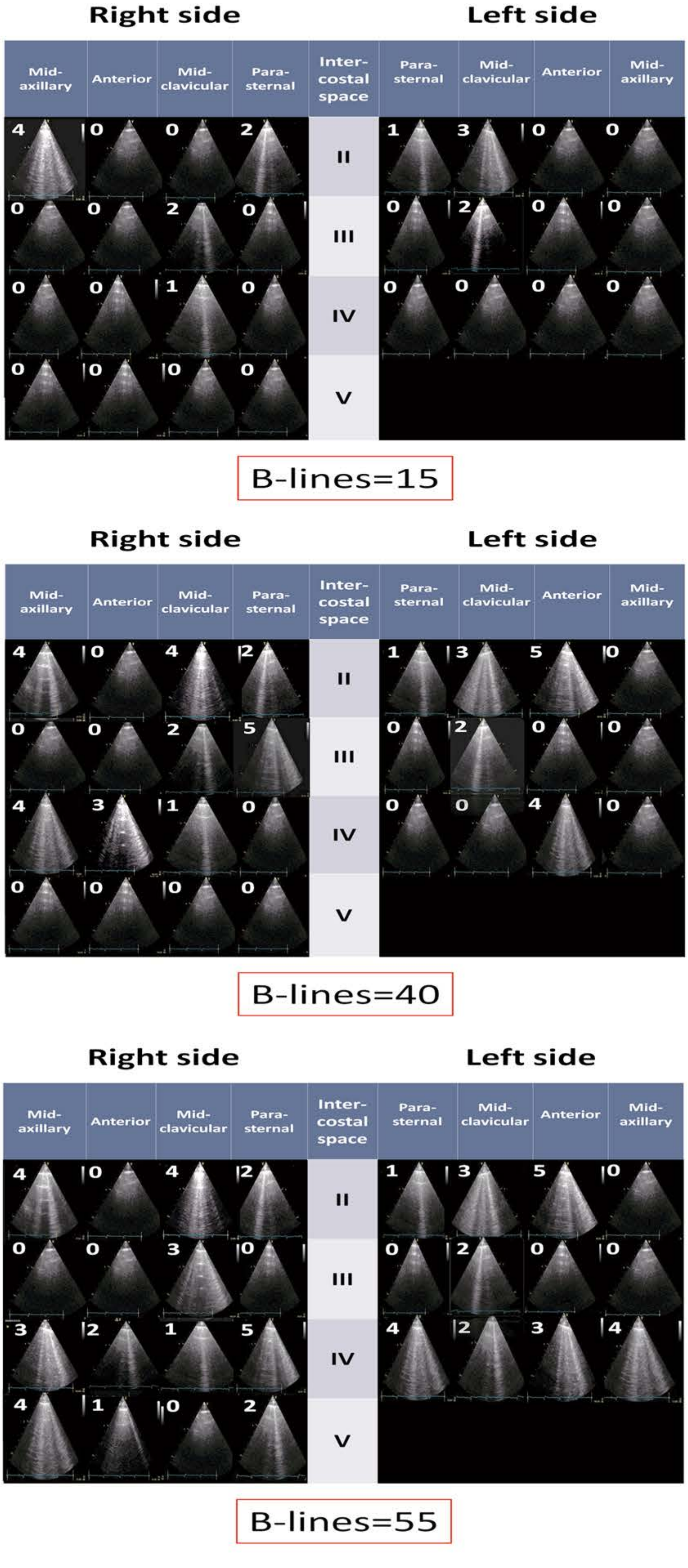
Right side
Left side
Mid-axillary
Anterior
Mid-clavicular
Para-sternal
Inter-costal space
Para-sternal
Mid-clavicular
Anterior
Mid-axillary
II
III
IV
V
B-lines=15
Right side
Left side
B-lines=40
Right side
Left side
B-lines=55

Supplementary Figure Legend

Supplementary figure: illustrative figures showing how to count B-lines.
